# Rapid Synthesis of Thin and Long Mo_17_O_47_ Nanowire-Arrays in an Oxygen Deficient Flame

**DOI:** 10.1038/srep27832

**Published:** 2016-06-08

**Authors:** Patrick Allen, Lili Cai, Lite Zhou, Chenqi Zhao, Pratap M. Rao

**Affiliations:** 1 Department of Mechanical Engineering, Worcester Polytechnic Institute, Worcester, MA 01609, USA; 2 Department of Mechanical Engineering, Stanford University, Stanford, CA 94305, USA; 3 Materials Science and Engineering Graduate Program, Worcester Polytechnic Institute, Worcester, MA 01609, USA

## Abstract

Mo_17_O_47_ nanowire-arrays are promising active materials and electrically-conductive supports for batteries and other devices. While high surface area resulting from long, thin, densely packed nanowires generally leads to improved performance in a wide variety of applications, the Mo_17_O_47_ nanowire-arrays synthesized previously by electrically-heated chemical vapor deposition under vacuum conditions were relatively thick and short. Here, we demonstrate a method to grow significantly thinner and longer, densely packed, high-purity Mo_17_O_47_ nanowire-arrays with diameters of 20–60 nm and lengths of 4–6 μm on metal foil substrates using rapid atmospheric flame vapor deposition without any chamber or walls. The atmospheric pressure and 1000 °C evaporation temperature resulted in smaller diameters, longer lengths and order-of-magnitude faster growth rate than previously demonstrated. As explained by kinetic and thermodynamic calculations, the selective synthesis of high-purity Mo_17_O_47_ nanowires is achieved due to low oxygen partial pressure in the flame products as a result of the high ratio of fuel to oxidizer supplied to the flame, which enables the correct ratio of MoO_2_ and MoO_3_ vapor concentrations for the growth of Mo_17_O_47_. This flame synthesis method is therefore a promising route for the growth of composition-controlled one-dimensional metal oxide nanomaterials for many applications.

To date, the synthesis of Mo_17_O_47_ (=MoO_2.76_) nanowire-arrays has only been reported by hot-filament chemical vapor deposition (CVD) under vacuum conditions^1^. These Mo_17_O_47_ nanowire-arrays demonstrated a high capacity retention of ∼630 mAhg^−1^ for up to 20 cycles at 50 mAg^−1^ current density as Li-ion battery anodes^1^, which is significantly larger than that achieved for MoO_3_ nanobelts^2^. The nanowire morphology resists agglomeration and provides a direct electrical conduction path to the current collector, while the Mo_17_O_47_ oxide composition provides high electrical conductivity due to the presence of large concentrations of oxygen vacancies^3^. In addition, high surface area (long, thin, densely packed nanowires) would also lead to improved performance. However, the Mo_17_O_47_ nanowire-arrays demonstrated so far had limited surface area due to relatively large diameters and short lengths. High-surface-area Mo_17_O_47_ nanowire-arrays would also be beneficial as active materials in battery cathodes and electrically-conductive supports in other electrochemical devices such as sensors, electrocatalysts, and in photoelectrochemical or photovoltaic devices. For instance, Mo_17_O_47_ pellets yielded the greatest reversible capacity among all substoichiometric molybdenum oxides as Li-ion battery cathodes^4^. Moreover, nanostructures composed of mixtures of reduced molybdenum oxides (MoO_x_, 2 < x < 3), of which Mo_17_O_47_ is one example, have recently shown enhanced performance not only as anodes in Li-ion batteries^1^,^3^,^5^, but also as supercapacitors^6^, organic LEDs/photovoltaics^7^, electron-optical MEMs^8^; and wear resistant^9^, photochromic^10^, and gas sensing^11^ materials, among others. Therefore, it is of great interest to grow high surface area Mo_17_O_47_ nanowire-arrays for these possible wide applications. Nonetheless, the selective synthesis of long, thin, densely-packed, high-purity Mo_17_O_47_ nanowire-arrays is a challenge.

Here, we report an alternative “flame vapor deposition” (FVD) method for the selective synthesis of longer, thinner, and densely packed high-purity Mo_17_O_47_ nanowire-arrays on a substrate. Compared to the nanowires previously synthesized by vacuum CVD, which had diameters of ~90 nm and lengths of approximately 1 μm after 30 minutes of growth, the present nanowires have diameters of 20–60 nm and lengths of 4–6 μm after 15 minutes of growth. The FVD synthesis is conducted at atmospheric pressure in the flow of hot gaseous combustion products from a flat CH_4_-air flame with no enclosing walls or chamber (Fig. 1)^12^,^13^,^14^,^15^,^16^,^17^. The synthesis relies on the flame to provide the heat and oxidizing gases required to evaporate and generate molybdenum oxide vapors from a solid molybdenum source that is placed above the flame. The vapors then deposit onto a temperature-controlled substrate and in a stagnation-point flow configuration. Compared to electrically-heated vacuum deposition methods, the atmospheric pressure and the relatively higher evaporation temperatures of the FVD synthesis allow the total concentration of MoO_x_ vapors to be larger, resulting in denser nucleation of nanowires with smaller diameters, and faster axial growth rates, leading to higher aspect ratio and surface area. In addition, a benefit of this approach is that the partial pressure of oxygen in the synthesis environment can be directly controlled over many orders of magnitude through control over the ratio of CH_4_ (fuel) and air (oxidizer) supplied to the flame^14^,^15^,^17^. This enables the synthesis of high-purity Mo_17_O_47_ nanowires as opposed to MoO_2_ or MoO_3_, by correct selection of the CH_4_/air flow ratio. This is in contrast to the hot-filament CVD approach, and most other vapor deposition approaches, in which control over the concentration of oxidizers is typically achieved by flowing or leaking oxidizing gases and controlling the total pressure with a vacuum system^1^,^18^,^19^. Finally, the flat flame in the FVD method can be scaled up for large-area deposition onto substrates, and the atmospheric operation of the FVD synthesis is less energy-intensive than maintaining a vacuum^13^,^14^,^15^,^16^,^17^.

The synthesis of molybdenum oxide nanostructures has previously been investigated using flames. The synthesis of MoO_2_ nanostructures in the form of rods with hollow channels by vapor deposition on a probe^20^,^21^,^22^,^23^, or elongated rectangular particles by vapor condensation in the gas phase^24^,^25^ has been achieved by evaporating Mo probes in or near the flame front in a counter-flow diffusion flame. MoO_3_ vapors were generated on the oxidizer size of the probe and were then converted to MoO_2_ and deposited on the fuel side. In addition, we have previously studied the variation of the fuel/air ratio, the Mo source temperature, and the substrate temperature using the FVD method for the synthesis of single, branched, and flower-like α-MoO_3_ nanobelts^16^, and studied the co-evaporation of Mo and W sources to produce W-doped α- and β-MoO_3_ nanobelts^13^. However, the present study is the first to demonstrate that the fuel/air ratio of the flame can be controlled to obtain high-purity Mo_17_O_47_ nanowires under specific oxygen-deficient (fuel-rich) conditions. Moreover, the chemical kinetics of the combustion reactions are analyzed through calculations using an established CH_4_-air combustion mechanism, to gain insight into the influence of the fuel/air ratio on the gas phase composition which leads to the synthesis of Mo_17_O_47_ instead of MoO_3_.

## Results

### Experiments

MoO_x_ nanostructures were synthesized by FVD directly on Ni and Mo foil substrates by oxidizing and evaporating Mo wires (see Methods). All experiments employed a deposition time of 15 minutes, a peak flame temperature of approximately 1100 °C, a Mo wire source temperature of approximately 1000 °C, and a substrate temperature of 570 °C. More detailed temperature measurements are given in the supplementary information, Table S1. In the flame synthesis method, the burner, flame and vapor source are operating at steady-state. Then, the substrates onto which the deposit occurs are introduced into the flame region at the start of the growth time, and are removed from the flame region at the end of the growth time. The insertion and removal process takes only about 1 second. Therefore, the time that the substrates spend in the flame region is 15 minutes, within an error of only a few seconds. Moreover, after it is inserted into the flame, the temperature of the substrate reaches 90% of the steady-state growth temperature within 2 seconds, and 99% of the growth temperature within 10 seconds, as measured by a thermocouple in contact with the substrate. The substrate temperature reported is the steady-state temperature.

The reaction between CH_4_ (fuel) and O_2_ (oxidizer) occurring in the flame is given by CH_4_ + 2O_2_ → CO_2_ + 2H_2_O for the stoichiometric case. An equivalence ratio (Φ) is commonly defined to compare the actual fuel-oxidizer ratio of the flame to its stoichiometric value, as given by equation (1).
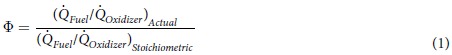
Here, 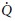 are the volumetric flow rates into the flame. Therefore Φ = 1 corresponds to a stoichiometric supply of CH_4_ and O_2_ into the flame, in which case the product gases will primarily consist of CO_2_ and H_2_O, while Φ > 1 corresponds to a fuel-rich flame, in which case the products will additionally contain unburned fuel in the form of H_2_ and CO, and Φ < 1 corresponds to a fuel-lean (oxygen-rich) flame, in which case the products will instead contain unreacted O_2_. The O_2_ in this experiment was supplied by flowing air, which contains approximately 21% of O_2_, and the same air flow rate of 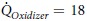 standard liters per minute (SLPM) was used for all experiments. Assuming that all the gases are ideal and have the same molar volumes, the stoichiometric flow rate of CH_4_ corresponding to this air flow rate is then 1.89 SLPM. The flow rate of CH_4_ was varied between experiments within the range of 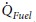 = 1.7 SLPM to 2.3 SLPM, which corresponds to a range of equivalence ratios of Φ = 0.90 (fuel lean) to 1.22 (fuel rich).

**Figure 1:**
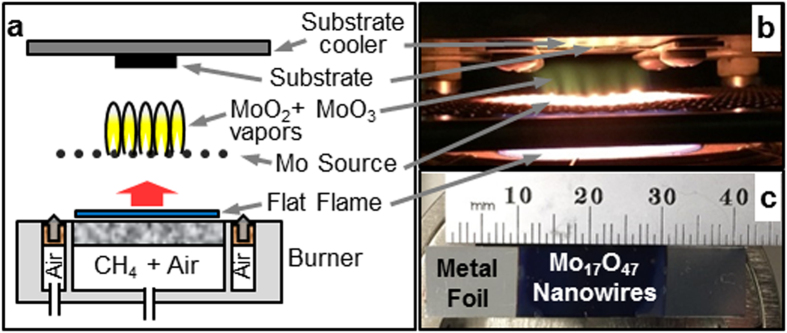
Description of the flame vapor deposition process. (**a**) Schematic and (**b**) photograph of flame vapor deposition setup. (**c**) Photograph of metal foil substrate with dark blue deposit consisting of Mo_17_O_47_ nanowire-arrays.

**Figure 2:**
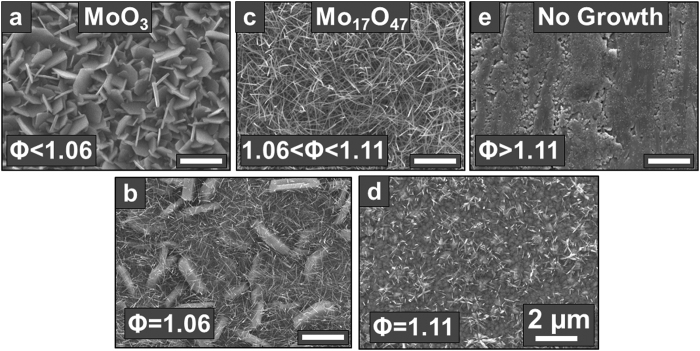
SEM images of the nanostructures grown on metal foils by flame vapor deposition at various equivalence ratios (Φ). (**a**) MoO_3_ nanobelts synthesized at Φ < 1.06, (**b**) a mixture of MoO_3_ nanobelts and Mo_17_O_47_ nanowires synthesized at Φ = 1.06, (**c**) long, thin, densely packed Mo_17_O_47_ nanowires synthesized at 1.06 < Φ < 1.11, (**d**) short, sparsely packed nanowires synthesized at Φ = 1.11, and (**e**) no deposit on the metal foil at Φ > 1.11. Scale bars in all images are 2 μm.

Scanning electron microscope (SEM) images of the resulting MoO_x_ nanostructures are shown in Fig. 2. At Φ < 1.06, MoO_3_ nanobelts were observed (Fig. 2a), which is consistent with the previous report^16^. As the fuel flow rate is increased to give Φ = 1.06, the morphology of the nanostructures transitions from MoO_3_ nanobelts to a mixture of nanobelts and nanowires (Fig. 2b). When Φ is further increased to 1.06 < Φ < 1.11, only densely packed nanowires are obtained (Fig. 2c). The loading density of the Mo_17_O_47_ was 0.6 +/− 0.1 mg/cm^2^ after 15 minutes of growth. This loading is comparable to that commonly used for nanowire electrodes in Li-ion battery research (~0.5 mg/cm^2^, 0.3 mg/cm^2^)^26^,^27^. At Φ = 1.11, the density and length of the nanowires decreases (Fig. 2d), and for Φ > 1.11, no deposit is observed (Fig. 2e). Visually, the growth of MoO_3_ nanobelts corresponds to a grey/white sample color, while the growth of nanowires corresponds to a blue/purple color (Fig. 1c). In both cases, the substrate surface is matte, while in the case of no deposit at high Φ, the surface is reflective. The temperature of the Mo source wires (from which the oxide vapor is being generated) increased by 8 °C from Φ = 0.95 to 1.16 (supporting information Table S1). This would result in increased generation of vapor from the Mo source, which cannot account for the change in morphology and composition of the deposited nanostructures from plate-like to wire-like, as this would instead be expected from a decrease in vapor. Moreover, we ensured that the substrate temperature of 570 °C was the same for all samples presented in Fig. 2, and was not influenced by any change in the flame temperature. This was done by carefully adjusting the water flow rate in the substrate cooler to compensate for any change in the flame temperature. Rather, the change in morphology of the nanostructures is primarily due to the change of oxygen partial pressure upon variation of Φ, as will be discussed later.

**Figure 3:**
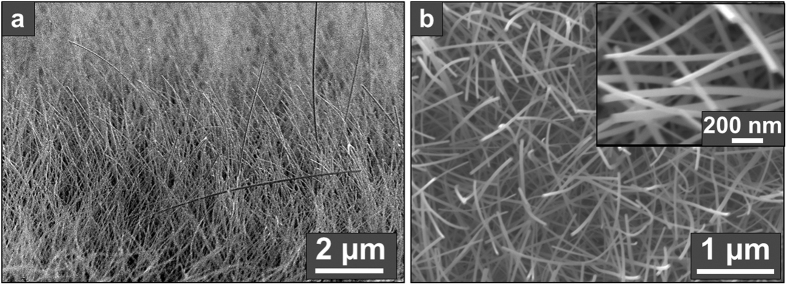
SEM images of Mo_17_O_47_ nanowire-arrays grown at 1.06 < Φ < 1.11. (**a**) Cross-section and (**b**) top-down SEM images of the Mo_17_O_47_ nanowire-arrays, which have diameters of 20–60 nm and lengths of 4–6 μm.

Higher-magnification side- and top-view SEM images of the nanowires grown at 1.06 < Φ < 1.11 are shown in Fig. 3. The nanowires have diameters of 20–60 nm and lengths of 4–6 um. The oriented nature of the nanowires demonstrates that they grow by the heterogeneous nucleation of vapors on the substrate and subsequent anisotropic growth of the nanowire crystals, rather than by homogeneous nucleation in the gas phase followed by deposition of the resulting particles, which would instead result in the deposition of a powder film of randomly-oriented crystals.

**Figure 4:**
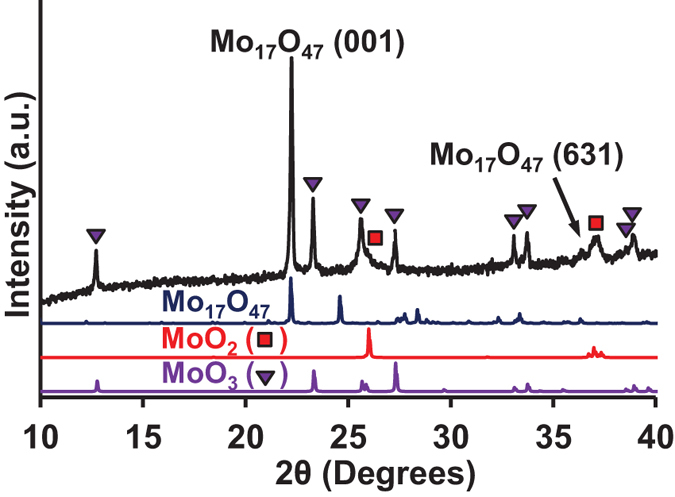
XRD pattern of the Mo_17_O_47_ nanowires synthesized on Ni foil, showing the presence of a dominant Mo_17_O_47_ phase, and also the presence of smaller quantities of MoO_2_ and MoO_3_ phases. Peaks from the Ni foil are present at larger angles.

X-ray diffraction (XRD) was performed on the nanowires grown at 1.06 < Φ < 1.11 on a Ni foil substrate to determine the crystal structure and composition of the nanowires. Comparison of the results to those of standard Mo oxides (MoO_2_, Mo_4_O_11_, Mo_5_O_14_, Mo_9_O_26_, Mo_17_O_47_ and MoO_3_), Mo-Ni oxides (NiMoO_4_), and Ni-oxides (NiO and NiO_2_) indicates that only orthorhombic Mo_17_O_47_ (ICDD PDF # 01-071-0566), monoclinic MoO_2_ (ICDD PDF # 04-003-1961), and orthorhombic MoO_3_ (i.e. α-MoO_3_, ICDD PDF # 04-015-7146) are present. Specifically, the results indicate the presence of a dominant Mo_17_O_47_ phase and smaller quantities of MoO_2_ and MoO_3_ phases (Fig. 4). The highest intensity of the signal from the Mo_17_O_47_ phase suggests that this signal comes from the nanowires, which according to SEM is the phase present in greatest quantity on the surface of the foil substrate. Moreover, because the relative intensity of the (001) reflection of Mo_17_O_47_ is much larger than that of the same peak in the powder standard, we can conclude that the Mo_17_O_47_ phase is strongly textured in the [001] direction, which would be consistent with Mo_17_O_47_ nanowires growing in the [001] crystal direction. The pattern attributed to the Mo_17_O_47_ phase also matches that obtained in a previous study on Mo_17_O_47_ nanowires synthesized by hot wire CVD^1^. In contrast, the MoO_2_ and MoO_3_ phases appear to have an isotropic texture because the relative intensities of the peaks in their signals match those of the powder standard. This suggests that they are present as polycrystalline layers, rather than part of the elongated nanowire crystals. The XRD signal from MoO_2_ persists even when the nanowires are scraped off the substrate, indicating that the MoO_2_ is present in an underlying layer formed by oxidation of the substrate during synthesis. As reported previously, the MoO_3_ signal can come from the oxidation of the surface of the Mo_17_O_47_ nanowires in air, after synthesis^1^. XRD analysis was also performed for the sample shown in Fig. 2e, which was obtained under the condition of Φ > 1.11. The result is given in supplementary information Fig. S1, and shows that there is no difference between the XRD pattern of the pristine substrate and that exposed to the flame synthesis, proving that there is no deposit in this case.

**Figure 5:**
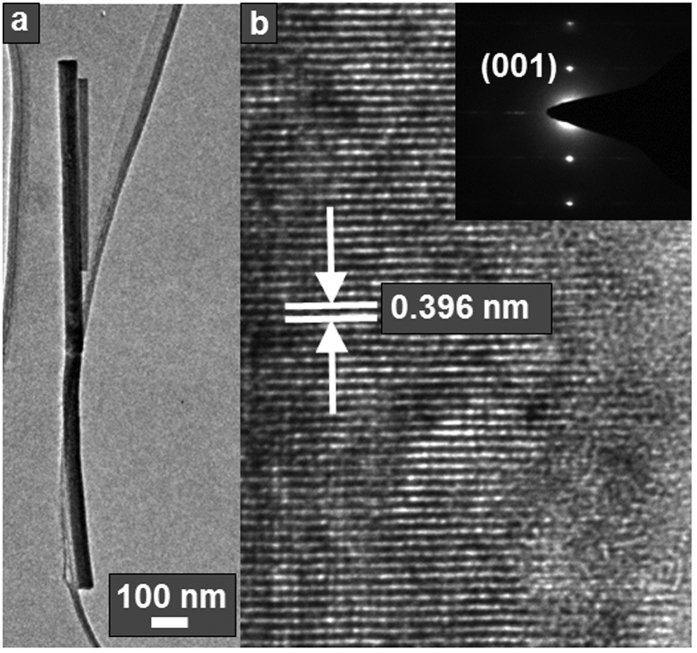
TEM analysis of the Mo_17_O_47_ nanowires. (**a**) Low-resolution TEM image of a Mo_17_O_47_ nanowire with the nanowire axis perpendicular to the electron beam and (**b**) high-resolution TEM image of the right edge of the nanowire showing lattice fringes that are perpendicular to the growth direction with spacing characteristic of the (001) planes of orthorhombic Mo_17_O_47_. The inset shows the corresponding SAED pattern, with the spots corresponding to diffraction from the (001) planes, and the streaks to the left and right of the spots resulting from disordered lattice spacings perpendicular to the (001) planes.

Transmission electron microscopy (TEM) was conducted to further investigate the crystal structure and composition of the nanowires grown at 1.06 < Φ < 1.11. The nanowires were removed from the growth substrate and dispersed onto a TEM grid. A nanowire such as that shown in Fig. 5a, which is oriented parallel to one of the two perpendicular tilt axes of the TEM stage, was selected. The nanowire was first tilted around the pitch axis to maximize the projected length of the nanowire in the TEM image, which serves to roughly orient the nanowire axis perpendicularly to the electron beam. The nanowire was then further tilted around the pitch axis until the crystal planes that are perpendicular to the nanowire axis became precisely parallel to the electron beam, as verified by selected area electron diffraction (SAED) (see diffraction pattern in the inset of Fig. 5b). High-resolution TEM (HRTEM) images of the crystal lattice were then obtained at this orientation, as shown in Fig. 5b. Both the HRTEM images and the SAED spot pattern show a lattice fringe spacing of 0.396 nm, which matches very well with the (001) plane spacing of the orthorhombic Mo_17_O_47_ (0.400 nm). To confirm that these (001) planes are indeed perpendicular to the nanowire axis, the nanowire was then rotated about its roll axis over a range of 60°, over which these planes remained parallel to the electron beam. Therefore, this confirms that the nanowires are composed of high-purity Mo_17_O_47_ with (001) crystal planes perpendicular to the nanowire axis. This also matches the findings of the XRD analysis, which showed higher relative intensity of the (001) reflection compared to the powder standard. The lattice spacings of planes parallel to the nanowire axis were more disordered, as indicated by the streaks to the left and right side of the bright spots in the SAED pattern.

The most distinct features of the Mo_17_O_47_ nanowires synthesized here by FVD are their small diameter, long length, and high packing density. The synthesis of Mo_17_O_47_ nanowire-arrays was previously reported by hot-filament CVD under vacuum conditions^1^. However, in that case, the nanowires had larger diameters of ~90 nm compared to the 20–60 nm diameters here, and larger lengths of approximately 1 μm (estimated from SEM images in that report) after a growth time of 30 minutes, compared to lengths of 4–6 μm after a growth time of 15 minutes here. These differences can be explained by the higher 1 atmosphere pressure and 1000 °C evaporation temperature of the FVD synthesis compared to the 1.1 Torr pressure and 775 °C evaporation temperature of the CVD synthesis, which results in higher pressure and supersaturation of MoO_x_ vapors in the FVD synthesis. The decrease of nucleus size with increasing supersaturation is well-known for heterogeneous nucleation of solids from vapor^28^,^29^, which leads to the smaller nanowire diameters observed here. At the same time, the higher vapor pressure leads to the faster axial growth rates observed here.

**Figure 6:**
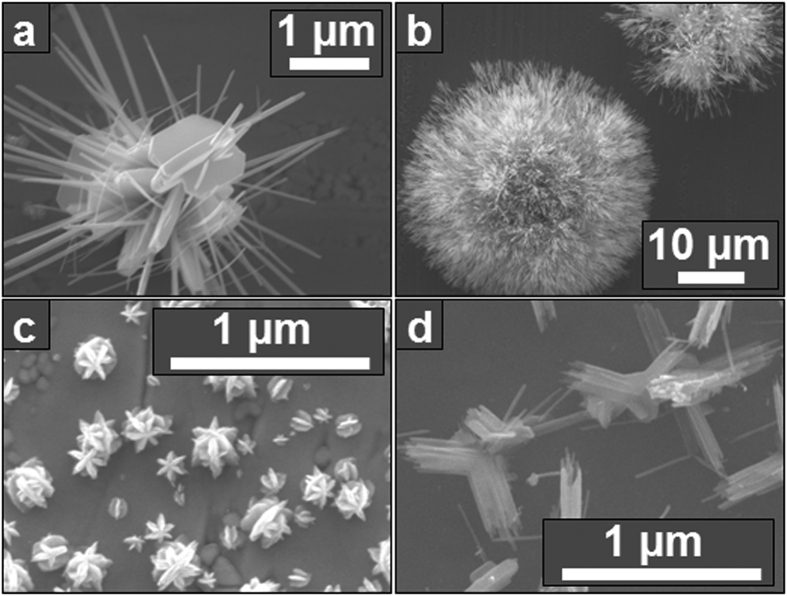
Unique morphologies grown by flame vapor deposition on metal foil substrates at temperatures below 500 °C. Mo_17_O_47_ nanowires growing on MoO_3_ nanobelts at (**a**) early stages and (**b**) later stages of growth. (**c**) Rounded MoO_3_ nanobelts growing perpendicularly from the faces of other MoO_3_ nanobelts. (**d**) Mo_17_O_47_ nanowires growing at right angles to one another with a common base.

Finally, it is noted that at Φ ≈ 1.1 but with a substrate temperature below 500 °C, unique structures with a mixture of morphologies and compositions are grown on the metal foils, as shown in Fig. 6. These structures may be further investigated in the future.

### Simulations

**Figure 7:**
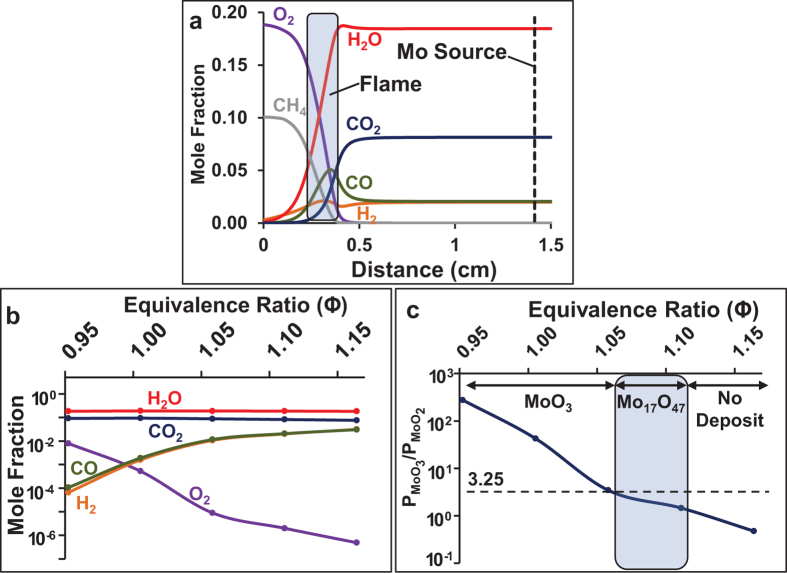
Simulations and calculations of the flame environment and molybdenum oxide vapor concentrations during flame vapor deposition. (**a**) Simulated species concentration profiles of O_2_, H_2_O, H_2_, CO_2_ and CO as a function of distance above the burner for the case of air flow rate of 18 SLPM and CH_4_ flow rate of 2.1 SLPM (Φ = 1.11). (**b**) Simulated species concentrations at a distance of 1.4 cm above the burner (the location of the Mo evaporation source) for various different equivalence ratios. (**c**) An estimate of the ratio of partial pressures of MoO_3_ and MoO_2_ vapors, 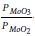, at various equivalence ratios assuming equilibrium of the reaction 2MoO_2_(g) + O_2_(g) → 2MoO_3_(g), for the O_2_ concentrations given in (**b**). The predicted 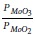 ratio is close to the value of 3.25 that is required for the stoichiometric deposition of Mo_17_O_47_ within the experimentally-observed equivalence ratio window of 1.06 < Φ < 1.11, and is much larger for lower Φ, which results in the deposition of MoO_3_ at Φ < 1.06.

Another key advantage of the FVD method is that the previously defined equivalence ratio (Φ) of the flame can be controlled to result in the growth of nanostructures composed of a pure phase (either Mo_17_O_47_ or MoO_3_) rather than mixtures of phases. The reason behind this composition control is studied by simulations of the chemical kinetics of combustion, and by thermodynamic calculations. The species concentration profiles as a function of distance above the burner were simulated using Chemkin PREMIX software^30^, employing the GRI-Mech 3.0 chemical kinetics mechanism for CH_4_ combustion^31^ and the experimentally measured gas temperatures. A representative result for the case of CH_4_ flow rate of 2.1 SLPM (Φ = 1.11) is shown in Fig. 7a. The results predict the formation of the flame a few millimeters away from the burner, which is consistent with the experiment, and also provide concentrations for relevant species such as O_2_, H_2_O, H_2_, CO_2_ and CO at the Mo evaporation source, which is located at 1.4 cm. The concentrations of these species are reported in Fig. 7b for various different equivalence ratios obtained from different simulations at different CH_4_ flow rates, keeping all else constant.

Data on the volatilization of Mo metal in the presence of oxidizers indicates that MoO_3_ and MoO_2_ molecules are evolved at the temperatures being studied here^32^. The transition from MoO_3_ growth at Φ < 1.06 to Mo_17_O_47_ growth at Φ > 1.06 can qualitatively be explained by a decrease in the oxidizing nature of the combustion products from the flame as Φ is increased, since at higher Φ the products contain greater fractions of reducing gases such as H_2_ and CO as opposed to oxidizing gases such as H_2_O, CO_2_ and O_2_. This should lead to a greater fraction of MoO_2_ vapors being generated compared to MoO_3_ vapors, therefore resulting in the growth of MoO_x_ oxides with 2 < x < 3. However, the oxidizing species are necessary for generating the MoO_2_ and MoO_3_ vapors in the first place by a process of simultaneous oxidation and evaporation of the surface of the solid Mo source wires. Studies on refractory metals such as Mo and W have shown that the rate of volatilization of the metal to produce gaseous oxides in the presence of O_2_ is orders of magnitude faster than that in the presence of the other oxidizers H_2_O and CO_2_, while at the same time, the presence of H_2_ and CO suppress volatilization^33^,^34^. Therefore, the lack of growth at Φ > 1.11 can be explained by the lack of generation of MoO_x_ vapors due to very low concentrations of O_2_ and increased concentrations of CO and H_2_ in the combustion products. Despite the large concentrations of H_2_O and CO_2_ at these equivalence ratios, these oxidizers are unable to oxidize Mo at the studied temperatures.

Once the MoO_2_ and MoO_3_ vapors are generated, the combustion products can further influence 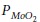 and 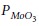, the partial pressures of MoO_2_ and MoO_3_ vapors, respectively, by reduction or oxidation in the gas phase. To estimate the ratio of partial pressures of these two species, we assume that the gas phase reaction 2MoO_2_(g) + O_2_(g) → 2MoO_3_(g) is at equilibrium because, as reported previously, the kinetics of oxidation by O_2_ are very rapid at these temperatures, while the kinetics of oxidation by H_2_O and CO_2_ are much slower^33^,^34^. Using the definition of the equilibrium constant for this reaction, we obtain equation (2), where 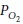 is the partial pressure of oxygen in atmospheres, *P*^0^ is the standard pressure of 1 atmosphere, Δ*G* is the change in Gibbs free energy for the reaction, *R* is the universal gas constant (8.314 J/mol.K), and *T* is the temperature in degrees Kelvin.
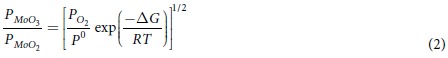


The thermodynamic data needed to evaluate Δ*G* were retrieved from the NIST Chemistry WebBook^35^. The resulting 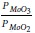 ratio is plotted in Fig. 7c for the oxygen concentrations given in Fig. 7b at the measured gas temperatures downstream of the Mo wires, which increased from 1051 to 1121 °C as Φ was increased from 0.95 to 1.16 (supplementary information Table S1). The final result is that the 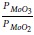 ratio decreases with increasing Φ because of a shift in the gas phase equilibrium of the exothermic reaction 2MoO_2_(g) + O_2_(g) → 2MoO_3_(g) towards MoO_2_. This shift happens primarily because the partial pressure of O_2_ decreases extremely sharply with equivalence ratio, and to a lesser extent because the temperature of the gas increases gradually with equivalence ratio in the range of interest.

The vapor has the same overall composition as the Mo_17_O_47_ nanowires when the 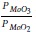 ratio is equal to 3.25. Therefore, if the MoO_3_ and MoO_2_ vapors condensed and were directly incorporated into the nanowires without further reaction, we would expect Mo_17_O_47_ nanowires to grow only when the 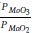 ratio equals 3.25. However, as shown in Fig. 7c, we instead observe that Mo_17_O_47_ nanowires grow when the 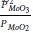 ratio has a range of values lower than 3.25, within the equivalence ratio window of 1.06 < Φ < 1.11. This can be explained if, after the MoO_2_ molecules adsorb onto the substrate or nanowires, some fraction of them undergo oxidation to MoO_3_ before being incorporated into the Mo_17_O_47_ nanowires by the reaction MoO_2_(ads) + ½ O_2_(ads) → MoO_3_(ads), in which “ads” denotes a species absorbed on the surface. The free energy for this reaction can be estimated by considering the free energy for the reaction MoO_2_(s) + ½ O_2_(g) → MoO_3_(s), which is Δ*G* = −98.74 kJ/mol at the substrate temperature of 570 °C and is highly spontaneous for O_2_ partial pressures above 5.8 × 10^−13 ^atm, which is the case for all conditions in this study. Therefore, if MoO_2_ vapors are in excess, MoO_2_ molecules can adsorb onto the substrate or nanowire surface, some of the molecules can be oxidized to MoO_3_ by adsorbed oxygen, and Mo_17_O_47_ nanowires can grow. On the other hand, if MoO_3_ is in excess, MoO_3_ molecules cannot be converted to MoO_2_ on the surface, and Mo_17_O_47_ does not grow. Indeed, as shown in Fig. 7c, we have observed that Mo_17_O_47_ nanowires grow only for Φ > 1.06 when the 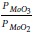 ratio is less than 3.25, that is, when MoO_2_ is in excess, and not when Φ < 1.06 when the 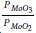 ratio is greater than 3.25, that is, when MoO_3_ is in excess. Rather, Φ < 1.06 results in the deposition of MoO_3_ instead. Taken together, these simulation results explain the chemical mechanism behind the control of the composition of the deposited oxide material via control of the flame equivalence ratio. Interestingly, other possible thermodynamically-stable MoO_x_ phases such as Mo_4_O_11_ and Mo_5_O_14_ were not found by XRD even though the 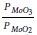 ratios for these phases are 3 and 4, respectively. This could be due to the faster nucleation or growth rate of the MoO_3_ and Mo_17_O_47_ nanowire crystals compared to crystals of other phases, although further experiments or simulations would be required to prove this.

## Discussion

We first discuss the origin of the anisotropic growth of the Mo_17_O_47_ crystals in the shape of elongated nanowires. From high-resolution SEM and TEM images, no distinct region, particles or other features were observed at the nanowire tips, suggesting that the growth occurs without the involvement of any chemically-distinct region at the tip of the nanowire as in the “vapor-liquid-solid” or “vapor-solid-solid” mechanisms^36^,^37^. Rather the growth appears to occur by adsorption of MoO_x_ vapor molecules onto the nanowire surface, followed by subsequent migration and incorporation of the molecules into the nanowire at the tip, which is the “vapor-solid” mechanism. As mentioned earlier, excess MoO_2_ can be oxidized to MoO_3_ on the nanowire or substrate surface by adsorbed oxygen, resulting in the growth of stoichiometric Mo_17_O_47_ nanowires. Although the reason for the nanowire shape of Mo_17_O_47_ is not presently known, we hypothesize that it may arise due to similar reasons as the nanowire shape of W_18_O_49_, which has a closely-related crystal structure. W_18_O_49_ nanowires are found to grow in the [010] direction perpendicular to their close-packed (010) planes^38^,^39^. A recent study found, using density functional theory calculations, that the (010) close-packed plane of W_18_O_49_ has high surface energy, while other low-index planes (001), (100) and (101) have lower surface energies^38^. The energy barrier for nucleating new layers onto a crystal is due to the formation of the sidewall surfaces of the new layer^40^. The energy barrier is therefore smallest for nucleation of new layers on the (010) plane of the W_18_O_49_ crystal since the sidewalls of the layer are composed of low-energy planes such as (001), (100) and (101). The small energy barrier results in fast nucleation of new layers on the (010) face, which then results in fast growth of the crystal in the [010] direction. Therefore, the anisotropic surface energies can explain the anisotropic structure of the W_18_O_49_ nanowires in terms of growth kinetics. The structure of Mo_17_O_47_ is very closely related to that of W_18_O_49_, with the (001) close-packed plane of Mo_17_O_47_ being structurally analogous to the (010) close-packed plane of W_18_O_49_, both containing distorted edge- and corner-sharing MO_6_ octahedra and MO_7_ pentagonal bipyramids in nearly the same arrangement^41^. Therefore, like the (010) plane of W_18_O_49_, we expect the structurally-analogous close-packed (001) plane of Mo_17_O_47_ to similarly have the highest surface energy among the low-index planes of Mo_17_O_47_, although this needs to be confirmed by calculations in the future. If true, the high surface energy of the (001) plane would adequately explain the fast growth of the Mo_17_O_47_ crystal in the [001] direction as a result of fast nucleation of new layers onto the (001) crystal face, resulting in the anisotropic wire structure of Mo_17_O_47_ with [001] axial direction as we have observed from TEM and XRD analyses (Figs 4 and 5).

Previous studies have investigated the vapor deposition of tungsten oxide nanowires by the generation of tungsten oxide vapors from a hot tungsten filament in the presence of O_2_ gas in a vacuum tube furnace, and the subsequent deposition of these vapors onto a substrate^42^,^43^. There are similarities between these prior studies and the present study, resulting from the chemical similarities between W, WO_2_ and WO_3_, and Mo, MoO_2_ and MoO_3_, which can generically be called A, AO_2_ and AO_3_. However, while these previous studies investigated a range of conditions in which the partial pressure of AO_2_ vapor is larger than that of AO_3_ vapor (i.e. P_AO3_/P_AO2_ < 1), the present study explores a range of conditions in which the partial pressure of AO_3_ is larger than that of AO_2_ (i.e. P_AO3_/P_AO2_ > 1), and additionally shows the transition of the growth from AO_3_ nanostructures (MoO_3_ nanobelts) at high P_AO3_/P_AO2_ to AO_2−x_ nanostructures (Mo_17_O_47_ = MoO_2.76_ nanowires) at lower P_AO3_/P_AO2_. In the previous studies, in which the vapor generated was primarily WO_2_ with smaller quantities of WO_3_, if the vapor molecules were incorporated into the nanowires at the same rates with which they adsorbed onto the surface, the observed tungsten oxide nanowire composition should have been similar to WO_2_. However, the actual tungsten oxide nanostructure composition was WO_3_ or W_18_O_49_ (=WO_2.72_). Therefore, it was hypothesized that tungsten oxide nanostructures were formed by reactions such as WO_2_(s) + 0.5 O_2_(g) → WO_3_(s) or WO_2_(s) + 0.36 O_2_(g) → W_18_O_49_(s)^42^,^43^. This process is similar to the possibility we have described in the present study, in which MoO_2_ vapors can adsorb and then undergo oxidation to MoO_3_ before being incorporated into the Mo_17_O_47_ nanowires. Another study similarly examined the vapor deposition of molybdenum trioxide (MoO_3_) nanowires and tubular structures by the generation of molybdenum oxide vapors from a hot molybdenum filament in the presence of O_2_ gas in a vacuum tube furnace, and the subsequent deposition of these vapors onto a substrate^44^, but did not provide analysis of the partial pressures or roles of MoO_2_ and MoO_3_, as has been done here. These prior studies on tungsten and molybdenum oxides additionally hypothesized that under the conditions of excess WO_2_ or MoO_2_, the deposited WO_2_ or MoO_2_ would lead to the formation of a “sub-oxide cluster”, which directs the one-dimensional (anisotropic) vapor-solid growth of the nanowires^42^,^44^. However, in the specific case of the present Mo_17_O_47_ nanowires, we do not observe any distinct region, particles or other features at the tips of the nanowires and instead hypothesize, as described above, that the anisotropic growth of the nanowires is expected from the anisotropy of the Mo_17_O_47_ crystal itself based on the fastest nucleation of new layers on the closest-packed (001) planes.

In conclusion, we have demonstrated a method to selectively grow long, thin, densely-packed, high-purity Mo_17_O_47_ nanowire-arrays using rapid atmospheric flame vapor deposition without any chamber or walls. High aspect-ratio (~100:1) Mo_17_O_47_ single-crystal nanowire-arrays were grown on Ni and Mo foils at axial growth rates of up to ~0.4 um/min, with diameters of 20–60 nm and lengths of 4–6 um. The atmospheric FVD growth and high evaporation temperatures achieved by the flame resulted in larger total concentrations of MoO_x_ vapors, which produced smaller diameters and faster axial growth rates compared to electrically-heated CVD synthesis under vacuum. As verified by chemical kinetics simulations, the concentrations of oxidizing and reducing gases in the synthesis environment were directly controlled over several orders of magnitude through changes in the CH_4_/air ratio of the flame, which in turn controlled the relative concentrations of MoO_2_ and MoO_3_ vapors to enable the deposition of high-purity Mo_17_O_47_ nanowires. This is a primary benefit of this approach over most other vapor deposition synthesis methods, in which control over the concentration of oxidizers is typically achieved by flowing or leaking oxidizing gases and controlling the total pressure with a vacuum system, which is more energy intensive. This study is the first to grow Mo_17_O_47_ nanostructures using a flame, whereas MoO_3_ and MoO_2_ have been previously demonstrated. The Mo_17_O_47_ nanowires synthesized here could find use as active materials in batteries, and as active materials or high-surface-area electrically conductive supports in other electrochemical devices such as sensors, electrocatalysts, and in photoelectrochemical or photovoltaic devices. Finally, the flame synthesis method that has been further developed here is a promising route for the growth of composition-controlled 1-D metal oxide nanomaterials for many applications. Moreover, due to the large deposition area, rapid growth rates, atmospheric pressure and chamber-less operation, this flame synthesis method may also have future promise in large-scale nanomanufacturing applications.

## Methods

### Nanowire Synthesis and Characterization

The Mo_17_O_47_ nanowires were synthesized using a 60 mm-diameter porous-plug co-flow premixed burner (flat-flame McKenna burner, Holthuis and Associates, Sebastopol, CA), which has been described in detail in previous work on flame vapor deposition (Fig. 1)^14^. The CH_4_-air flame forms a flat 2-dimensional sheet above the burner surface. The flows of CH_4_ and air are delivered to the burner by calibrated rotameters (Brooks Instrument). The air flow rate was fixed at 18 SLPM for all experiments, while the CH_4_ flow rate was varied over the range of 1.7 SLPM to 2.3 SLPM for successive experiments. A constant flow of 22 SLPM air was also fed through a co-annular ring that surrounds the burner to ensure smooth flow by matching the velocity of the combustion product gases at the edge of the burner. The gas temperature in the post-flame region was maintained at approximately 1100 °C. The Mo evaporation source, which consists of five segments of 37 mm-length Mo wire (0.5 mm diameter, annealed, 99.95% purity, Alfa Aesar), is held above the flame by a plain steel mesh (0.254 cm wire spacing, 0.0635 cm wire diameter, McMaster-Carr) and is heated to a constant temperature of approximately 1000 °C. The surface of these Mo wires is continuously oxidized and evaporated during the synthesis to generate the MoO_x_ vapors. The vapors are convected upwards by the flow and deposit onto the substrate, which is either Ni foil (0.127 mm thick, 1 cm × 4.5 cm, 99.9% purity, annealed, Alfa Aesar) or Mo foil (0.05 mm thick, 1 cm × 4.5 cm, 99.95% purity, Alfa Aesar). The substrate is clamped to an internally water-cooled substrate holder (Al plate, 4-pass Cu tube, Lytron Model CP10G14), which is used to position the substrate and control its temperature. The vapor source (Mo wires) and the substrate are centered with respect to the centerline of the burner at heights of 14 mm and 29 mm, respectively, above the top surface of the burner. The temperature of the substrate is controlled by the flow rate of cooling water through the substrate holder (0.265 SLPM), as well as by controlling the distance between the substrate and the holder through the addition of 0.127 mm-thick stainless steel spacers placed between the clamp and the substrate. Temperatures were measured with a K-Type thermocouple (0.158 cm exposed bead, XL sheath, Omega Engineering Inc.). The nanowires were characterized by SEM (JEOL JSM-7000F, 10 kV), XRD (PANalyticalXPert 2, Cu-kα, 45 kV, 40 mA), and TEM (FEI Tecnai G2 F20 X-TWIN FEG).

### Combustion Kinetics Simulations

The species concentration profiles as a function of distance above the burner were simulated using Chemkin PREMIX software^30^, employing the GRI-Mech 3.0 chemical kinetics mechanism for CH_4_ combustion^31^ and the experimentally measured gas flow rates and gas temperatures.

## Additional Information

**How to cite this article**: Allen, P. *et al*. Rapid Synthesis of Thin and Long Mo_17_O_47_ Nanowire-Arrays in an Oxygen Deficient Flame. *Sci. Rep.* **6**, 27832; doi: 10.1038/srep27832 (2016).

## Supplementary Material

Supplementary Information
