# Supplementary material for: Rapid Synthesis of Thin and Long Mo17O47 Nanowire-Arrays in an Oxygen Deficient Flame
Source: Sci Rep. 2016 Jun 8;6:27832. doi: 10.1038/srep27832 (PMC4897683; doi:10.1038/srep27832)
Supplement: Supplementary Information [file srep27832-s1.doc]

**Rapid Synthesis of Thin and Long Mo_17_O_47_ Nanowire-Arrays in an Oxygen Deficient Flame**

**Supplementary Information**

Patrick Allen^1^, Lili Cai^3^, Lite Zhou^1,2^, Chenqi Zhao^1,2^ and Pratap M. Rao^1,2^*

^1^Department of Mechanical Engineering, Worcester Polytechnic Institute, Worcester, MA 01609, USA

^2^Materials Science and Engineering Graduate Program, Worcester Polytechnic Institute, Worcester, MA 01609, USA

^3^Department of Mechanical Engineering, Stanford University, Stanford, CA 94305, USA

*Correspondence and requests for materials should be addressed to P. M. R. (email: pmrao@wpi.edu)

| **Equivalence Ratio (Φ)** | **Temperature of Flame (°C)** | **Temperature of Mo wires (°C)** | **Gas temperature 0.5 cm downstream of Mo wires (°C)** |
| --- | --- | --- | --- |
| 0.95 | 1086 | 967 | 1051 |
| 1.01 | 1107 | 970 | 1074 |
| 1.06 | 1126 | 972 | 1096 |
| 1.11 | 1136 | 972 | 1101 |
| 1.16 | 1170 | 975 | 1121 |
| 1.22 | 1162 | 973 | 1117 |

**Table S1.** Detailed temperature measurements of the flame, the Mo wires, and the gas temperature downstream of the Mo wires, at different equivalence ratios (Φ).


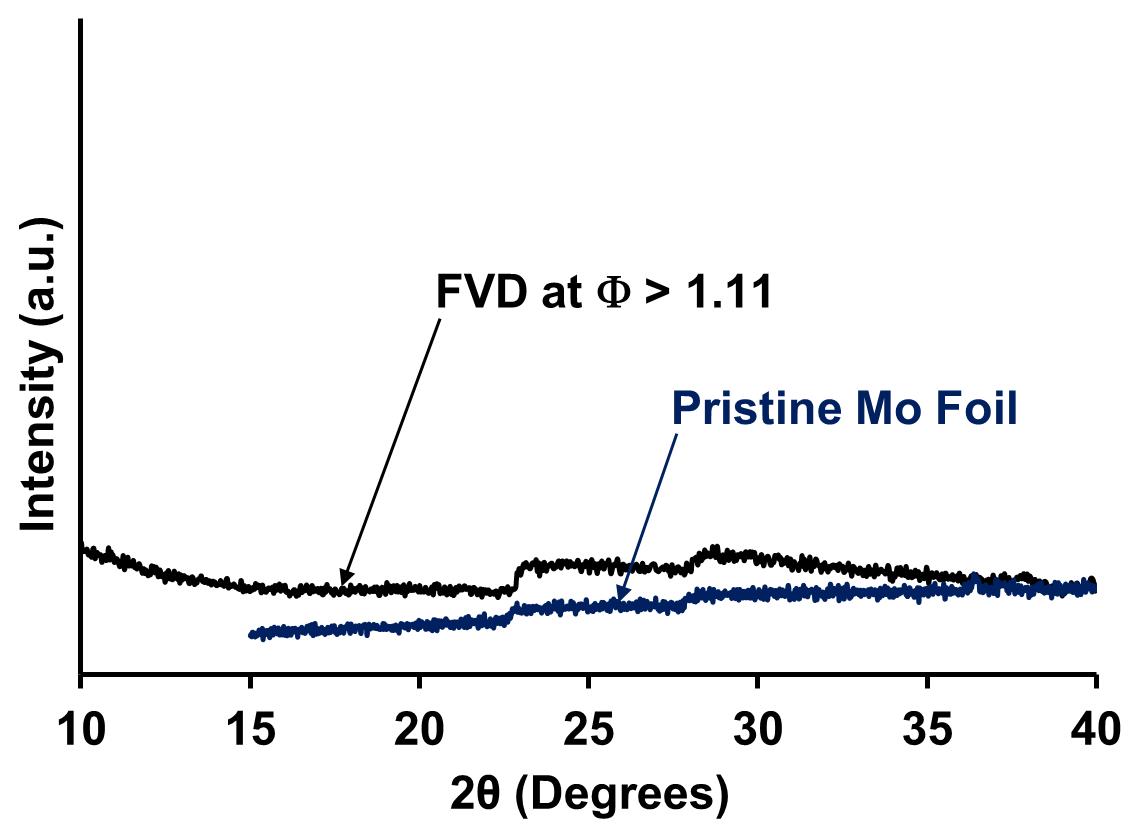


**Figure S1.** XRD patterns of pristine Mo foil and Mo foil exposed to flame vapor deposition (FVD) at Φ > 1.11. The x- and y- axis ranges are the same as in Figure 4 in the main text.
